# A novel missense mutation in the *HSF4* gene of giant pandas with senile congenital cataracts

**DOI:** 10.1038/s41598-021-84741-5

**Published:** 2021-03-08

**Authors:** Yuyan You, Chao Bai, Xuefeng Liu, Maohua Xia, Yanqiang Yin, Yucun Chen, Wei Wang, Ting Jia, Yan Lu, Tianchun Pu, Chenglin Zhang, Xiaoguang Li, Liqin Wang, Yunfang Xiu, Lili Niu, Jun Zhou, Yang Du, Yanhui Liu, Suhui Xu

**Affiliations:** 1 Beijing Key Laboratory of Captive Wildlife Technologies, Beijing Zoo, Beijing, China; 2 Beijing Zoo, Beijing, China; 3 Chongqing Zoo, Chongqing, China; 4 Strait (Fuzhou) Giant Panda Research and Exchange Centers, Fuzhou, China; 5 Chengdu Zoo, Chengdu, China

**Keywords:** Mutation, Molecular biology

## Abstract

Cataracts are a common cause of visual impairment and blindness in mammals. They are usually associated with aging, but approximately one third of cases have a significant genetic component. Cataracts are increasingly prevalent among aging populations of captive giant pandas (*Ailuropoda melanoleuca*) and it is therefore important to identify genetic determinants that influence the likelihood of cataract development in order to distinguish between congenital and age-related disease. Here we screened for cataract-related genetic effects using a functional candidate gene approach combined with bioinformatics to identify the underlying genetic defect in a giant panda with congenital cataracts. We identified a missense mutation in exon 10 of the *HSF4* gene encoding heat shock transcription factor 4. The mutation causes the amino acid substitution R377W in a highly conserved segment of the protein between the isoform-specific and downstream hydrophobic regions. Predictive modeling revealed that the substitution is likely to increase the hydrophobicity of the protein and disrupt interactions with spatially adjacent amino acid side chains. The mutation was not found in 13 unaffected unrelated animals but was found in an unrelated animal also diagnosed with senile congenital cataract. The novel missense mutation in the *HSF4* gene therefore provides a potential new genetic determinant that could help to predict the risk of cataracts in giant pandas.

## Introduction

Cataracts are eye defects in which the lens becomes cloudy and eventually opaque. Most cataracts are associated with aging, and are thought to reflect cumulative oxidative damage that progressively disrupts the reducing environment of the lens. This causes the accumulation of pigments and/or the aggregation of crystallin proteins, which are normally transparent and confer refractive properties^1^. Approximately one third of cataracts have a significant genetic component, in some cases due to the disruption of normal lens development and in others due to mutations in the crystallin proteins themselves or in other proteins required for normal lens physiology, including the oxidative stress response pathway^2^.

Cataracts are a prevalent cause of visual impairment and blindness in humans, other primates, and companion animals, so most cataract research has focused on these species, or on murine disease models^2^. However, cataracts are also very common in zoo animals, which tend to live longer than their wild counterparts and thus suffer from age-related diseases to a degree not seen in the wild. For example, more than 20% of the aged population of giant pandas (*Ailuropoda melanoleuca*) in China suffers from cataracts, which has a significant effect on their quality of life^3^. The development of preventative strategies and treatments for cataracts in captive pandas therefore requires more research into the congenital and age-related forms of this disease^3^.

Heat shock transcription factor 4 (HSF4) is associated with several forms of congenital cataract in humans, typically with an autosomal dominant inheritance pattern^4,5^. The human *HSF4* gene was identified by screening a HeLa cell cDNA library using the chicken *HSF3* gene as a probe^6^ and was subsequently mapped to a locus on chromosome 16 already associated with congenital cataracts^5,6^. The *HSF4* gene responds to various forms of stress and the corresponding protein acts as a transcriptional repressor to protect cells against proteotoxic damage^6^. HSF4 is also involved in the regulation of differentiation and development, and the isoform HSF4b (produced by alternative splicing) is predominantly expressed in the lens^7,8^. Although congenital cataracts associated with *HSF4* mutations were initially identified as autosomal dominant traits, others are autosomal recessive, indicating that different types of mutation may have different effects on cataract development^5,9–13^. At the molecular level, *HSF4* mutations appear to promote lens de-nucleation and thus the loss of lens protein function^14^. Furthermore, some *HSF4* mutations promote the development of age-related cataracts rather than highly penetrant congenital cataracts^15,16^.

A number of animal models have been identified with HSF4-related congenital cataracts, including the autosomal recessive mouse mutant Lop11^17^ and a spontaneous mutation in dogs^18^. However, similar mutations have not been reported in the giant panda. Here we used a functional candidate gene screening approach to test known cataract-associated genes in giant panda specimens with and without cataracts. We identified a novel missense mutation in *HSF4* affecting a female giant panda whose cataracts began to form at 28 years of age which was absent from 13 unaffected controls but present in another panda with cataracts. This will help to clarify the relationship between mutations in candidate genes and the prevalence of cataracts in pandas, providing more efficient cataract risk assessment for giant pandas in captivity.

## Results

### Clinical findings

**Figure 1:**
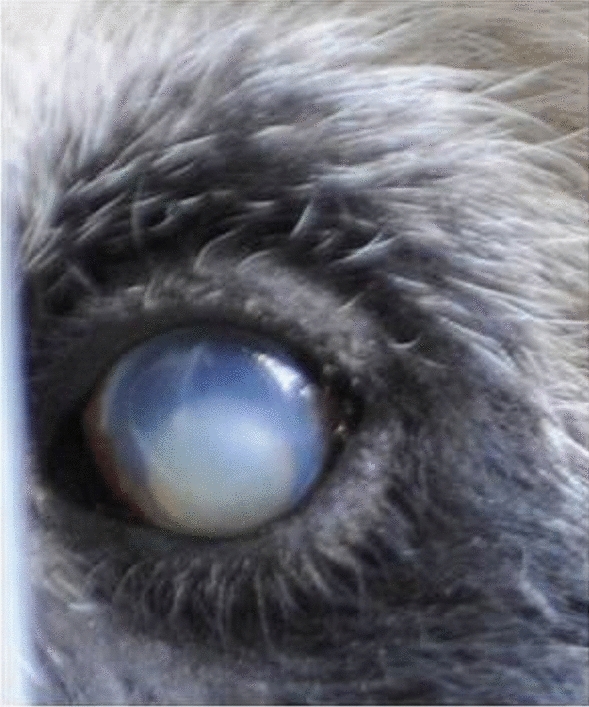
The left eye of Xinxing, a female giant panda with hyper-mature cortical cataracts.

Xinxing (the proband in this study, designated S4 in Table S1) underwent a clinical examination at the age of 28 and was diagnosed with hyper-mature cortical cataracts. Specifically, the cataracts were characterized by lens capsule shrinkage, deepening of the anterior chamber, a sunken lens nucleus (morgagnian cataract), deposition of lens cortical particles in the anterior chamber angle, trabecular meshwork plugging, secondary glaucoma (lens-induced glaucoma), a turbid lens dislocated into the anterior chamber, and corneal leukoplakia (Fig. 1). The hardness of the lens nucleus (emery) was determined as grade II. Xinxing has no history of related systemic abnormalities. Xinxing was born in the wild in Baoxing County, hence samples and medical histories are unavailable from her parents or any potential siblings. Xinxing has two known offspring, one of which was released into the wild in 2008 and the other exported to a zoo outside China, hence samples and medical histories are unavailable from these individuals either. In the absence of pedigree samples, we obtained samples from 14 unrelated individuals from diverse geographical regions of China (Beijing, Baoxing, Ya’an, Wolong, and Chengdu). The group comprised seven females and seven males, 13 of which were healthy at the time of sampling. One of the male donors (S1) was diagnosed with hereditary cataracts during regular medical examinations, although the cataracts were not present in the young animal and manifested with aging, but his female offspring (S2) did not suffer any cataract symptoms up to the time of her death. This group of genetically diverse pandas was sampled to ensure that any disease-associated mutations we discovered were genuine, which may disease associated genetic variant.

### Mutation analysis

**Figure 2:**
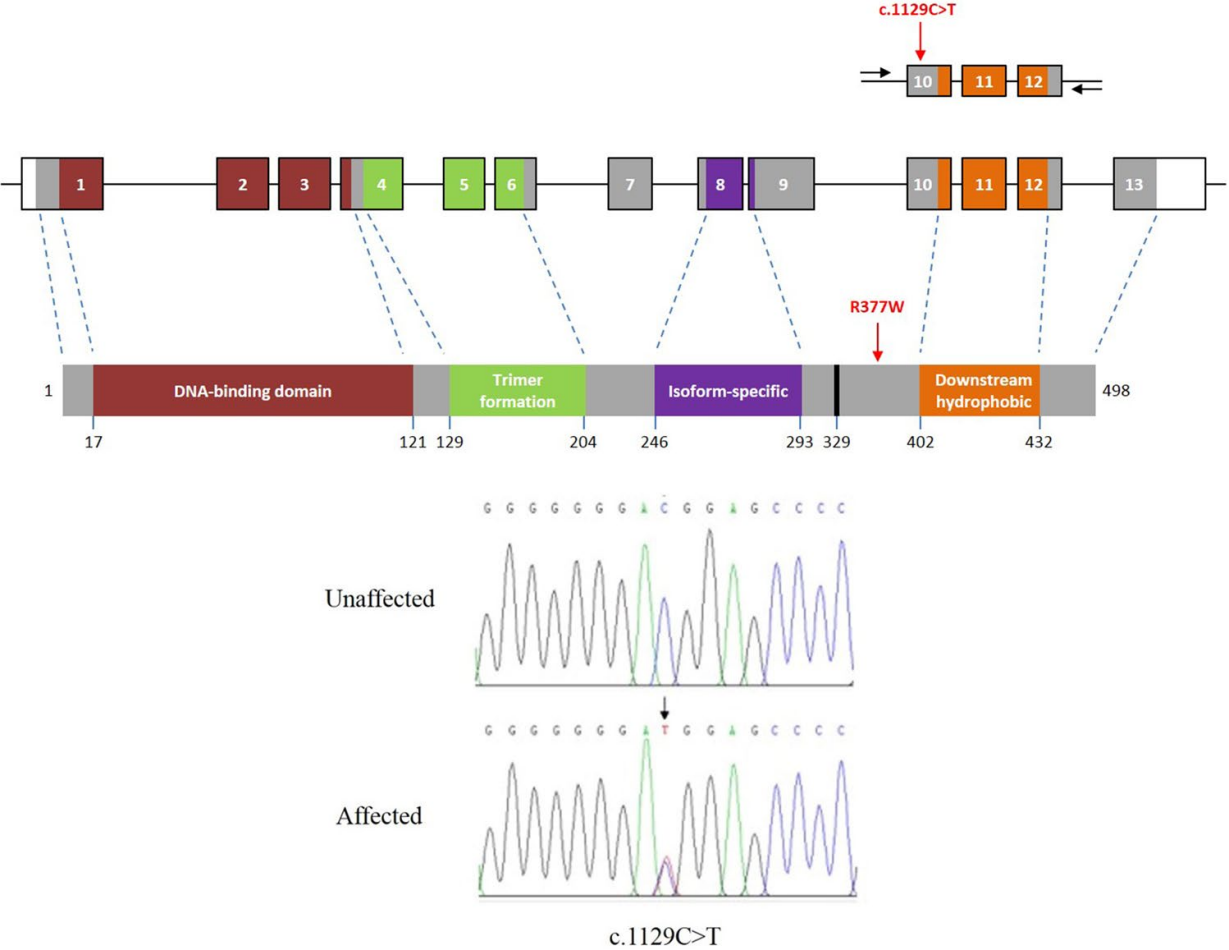
Location and identification of the HSF4 mutation in Xinxing. Structure of the panda HSF4 gene and the corresponding protein. Exons 10–12 (the region spanned by the PCR primers used to detect the mutation) are expanded, with exons shown as open boxes, introns shown as lines, primer positions shown as black arrows and the position of the mutation indicated with a red arrow. The black bar at position 329 indicates the glycine-rich segment specific to panda HSF4.

Genomic DNA was extracted from the blood samples of Xinxing (S4) and the 14 unrelated specimens and screened for mutations in 11 candidate genes associated with cataracts in humans and other mammals (Table S2). The comparison of PCR products from Xinxing and the 13 healthy control specimens revealed a novel c.1129C>T missense mutation in exon 10 of the *HSF4* gene (Fig. 2), which replaced the arginine residue at position 377 with a tryptophan residue (p.R377W). Remarkably, the same mutation was also identified in S1, the only unrelated panda diagnosed with cataracts, but was not present in his unaffected daughter S2. The mutation was the only sequence difference unique to the affected individuals, and the heterozygous nature of the mutant allele suggested that c.1129C>T is a pathogenic mutation that is inherited in an autosomal dominant manner.

### Sequence analysis and structural modeling

**Figure 3:**
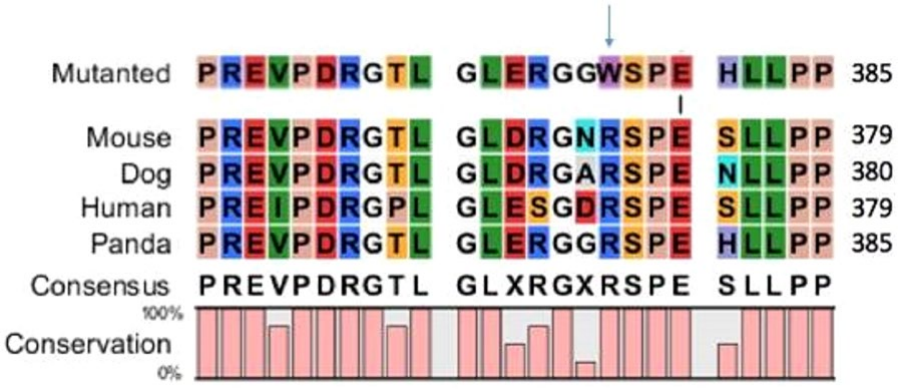
Effect of replacing a highly-conserved arginine residue with tryptophan between the isoform-specific and downstream hydrophobic regions of HSF4. Multiple alignment of a highly-conserved sequence of 25 amino acid residues in four orthologs of HSF4 (mouse, dog, human and panda) showing that the panda R377W substitution affects an arginine residue conserved across all species (equivalent to human residue R371).

**Figure 4:**
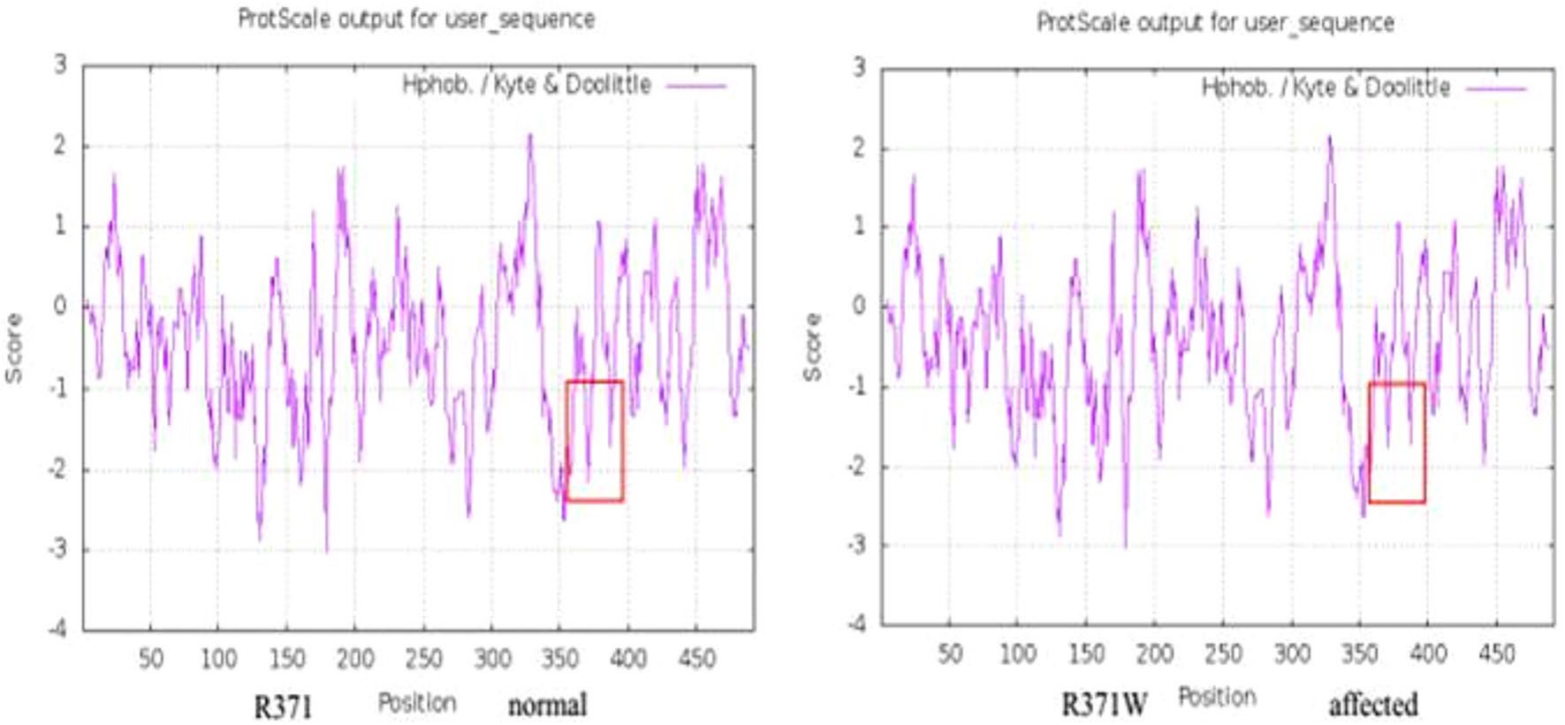
ProtScale analysis of the human protein with the equivalent mutation (R371W) confirming an increase in overall hydrophobicity.

To determine the importance of the R377W substitution for the structure and function of HSF4, we aligned the giant panda HSF4 sequence with its human, mouse and canine orthologs. This revealed that the R377W mutation is located in a highly-conserved region spanning residues 362–386, with the substitution affecting an arginine residue that is fully conserved in all four species (Fig. 3). The strong conservation of the arginine residue suggests that its positively-charged side chain plays an important role in the structure and/or function of HSF4, which is likely to be disrupted when replaced with the more bulky and hydrophobic tryptophan residue. ProtScale analysis of the human HSF4 protein confirmed that a corresponding mutation (R371W) would cause an increase in overall hydrophobicity that would influence how HSF4 binds to its interaction partners (Fig. 4).

**Figure 5:**
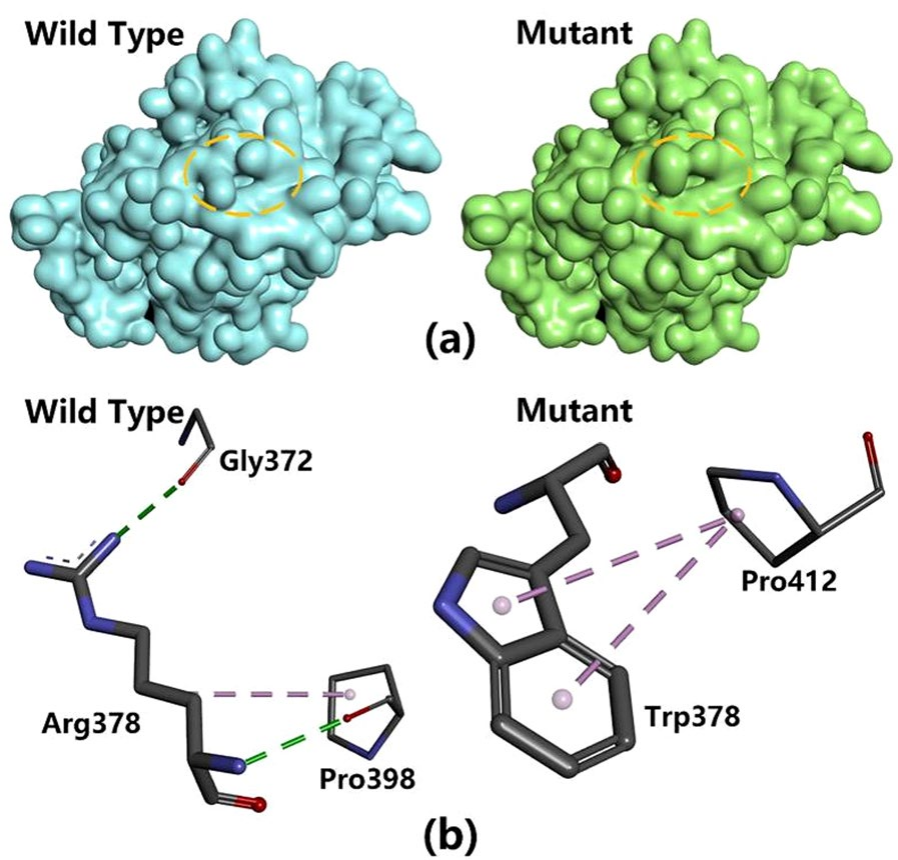
Predicted structural changes based on the missense mutation R377W in the giant panda HSF4 protein. (**a**) Changes in surface structure visualized using Discovery Studio Visualizer. (**b**) Interactions between amino acid side chains predicted using THREADER and Modeller.

We therefore used Modeller and THREADER to predict how the R377W substitution might affect the tertiary structure of HSF4, with *Streptococcus pneumoniae* hyaluronate lyase (PDB: 1N7O) as the template. The predicted changes on the surface of the protein are visualized in Fig. 5a. In the wild-type protein, the R377 residue forms hydrogen bonds with G372 and P398, and one cation–π interaction with P398 (Fig. 5b, left). In contrast, the replacement W377 residue engages in two π–π stacking interactions with P412 (Fig. 5b, right). The functional impact of these structural changes is unclear, but the segregation of the mutant allele in the two affected pandas and the wild-type allele in the 13 healthy controls indicates a strong correlation between the mutation and the propensity to develop cataracts.

## Discussion

Congenital cataracts are caused by mutations that disrupt lens development or normal lens physiology, including the weakening of stress response pathways that protect the lens from oxidative damage^1,2^. Some mutations affect the crystallin proteins that maintain lens transparency, whereas others affect structural proteins of the connexin and myelin precursor families, gap junction proteins, or stress response components^19^. One particularly interesting group of mutations affect transcription factors that are expressed in the lens, such as FOXE3, HSF4, MAF and PITX3, because these have the potential to disrupt the activity of many downstream genes^20,21^. Cataracts are a prevalent cause of visual impairment and blindness in many vertebrates, including captive zoo animals such as giant pandas. We recently investigated the genetic basis of age-related cataracts in giant pandas by MethylRAD sequencing, but it is important to find genetic markers that can specify the congenital and sporadic (age-related) forms of this disease^3^.

In order to identify genes associated with congenital cataracts in the giant panda, we selected a panel of 11 candidate genes that are commonly associated with human congenital cataracts and screened them systematically for mutations using an exon-scanning PCR approach. We identified a mutation in exon 10 of the *HSF4* gene encoding heat shock transcription factor 4 (HSF4), a protein associated with several forms of autosomal dominant congenital cataract in humans^4,5^ that has also been implicated as a cause of age-related cataracts^4,9,16,22^. *HSF4* encodes a transcriptional regulator that is needed for normal lens development and fiber cell differentiation^8,23,24^. Target genes have been identified by comparing gene expression profiles in the lenses of wild-type mice and homozygous *hsf4*^–/–^ knockouts^25,26^ revealing the suppression of multiple crystallin genes in various genetic backgrounds^7,27,28^. HSF4 also induces lens fiber cell differentiation by activating p53 and its downstream regulators, resulting in early-onset cataracts in *hsf4*^–/–^null zebrafish^8^. Different mutations therefore appear to have different clinical effects and it is important to characterize specific mutations in more detail to understand the link between each mutation and the manifestation of cataract disease.

A recent literature survey summarized 16 studies that reported a total of 14 unique, disease-causing mutations in the human *HSF4* gene, most of which were missense mutations, as well as individual cases of frameshift and nonsense mutations^21^. A homozygous splicing mutation associated with autosomal recessive congenital cataracts has also been described^10^. Interestingly, most of the mutations occurred in the DNA-binding domain of HSF4, as well as some in the oligomerization domain required for the formation of trimers or in the downstream hydrophobic repeat. However, no disease-causing mutations have been reported in the isoform-specific region unique to HSF4b, or in the large conserved block of amino acids between the isoform-specific region and the downstream hydrophobic repeat. Additional disease-causing mutations have been found in reports published after the literature survey, but these are also located primarily in the DNA-binding domain^28–30^ and one very near to the C-terminus of the protein^31^. The discovery of a pathogenic mutation between the isoform-specific and downstream hydrophobic regions of the giant panda HSF4 protein therefore indicates a potential novel disease mechanism.

The heterozygous c.1129C>T mutation we detected in Xinxing and the unrelated male S1 causes the substitution R377W, which replaces an arginine residue that is fully conserved in all the mammalian orthologs we analyzed. This residue lies downstream of the region specific isoform HSF4b, the main splice variant expressed in the lens, and within a highly-conserved segment just before the downstream hydrophobic region. The equivalent human mutation (R371W) has not been reported to our knowledge, although multiple studies have described pathogenic mutations in other parts of the human protein caused by the replacement of arginine residues, including R73H^9,15^, R73L^28^, R110C^32^, R119C^5,33^, R119H^30^, and R175P^11^. Almost all of these mutations affect the DNA-binding domain, with only R175P found in the oligomerization domain^11^. Finally, a nonsense mutation affecting an arginine residue in the downstream hydrophobic repeat (R405X) has also been reported^12^. Given the lack of pathogenic human mutations in this region, we cannot predict the pathogenetic mechanism in pandas, but our in silico analysis of the equivalent human mutation (R371W) revealed an increase in hydrophobicity caused by the replacement of arginine with tryptophan, which is likely to affect the binding of HSF4 to its interaction partners even if its DNA-binding capacity and ability to form trimers is unaffected. One potential reason for the absence of an equivalent mutation in humans is that the panda R377W mutation lies just downstream of a glycine-rich insert (GGGAPRG) which is, as far as we can tell, unique to the giant panda and not even present in closely related species such as the grizzly bear (*Ursus arctos horribilis*). The presence of multiple glycine resides and a single proline residue is likely to have a profound effect on the folding of the polypeptide backbone compared to orthologs lacking this sequence, and the effect of a nearby mutation that converts arginine into tryptophan may therefore be influenced by the context of the glycine-rich sequence. HSF4 is known to interact with partners such as BCL6^34^, dual specificity phosphatase 26 and several members of the mitogen-activated protein kinase (MAPK) family^35^, Brg1/SMARCA4^36^, ALKBH4^37^, DAXX^38^, HIF-1α/HSF2^39^ and others identified in large-scale interaction screens^40^. Therefore it is possible that the pathogenetic mechanism may involve the disruption of interactions with one or more of these regulators.

The replacement of the positively charged arginine residue with bulky tryptophan is also likely to change the surface properties of the protein (Fig. 5a) and to influence the formation of hydrogen bonds. We predicted that R377 in the wild-type protein is likely to form hydrogen bonds with G372 and P398, and a cation–π interaction with P398, but that tryptophan in the same position would instead engage in two π–π stacking interactions with P412 (Fig. 5b). A change in surface properties was also predicted for the R73L mutation in the human HSF4 protein^28^, and although no changes in H-bond formation were predicted for this particular mutant, new hydrogen bonds were predicted for the mutations Y78C, S105T and F63L^28^. It is therefore clear that mutations affecting the surface topography and intramolecular chain interactions within the HSF4 protein are associated with cataract disease, and we propose that the novel R377W mutation is likely to elicit a similar mechanism in giant pandas.

In summary, cataracts affect a large proportion of aging giant pandas in captivity but it is important to distinguish age-related cataracts from congenital disease caused by mutations in genes such as *HSF4*. We identified a novel mutation in exon 10 of the panda *HSF4* gene which was uniquely found in two affected pandas and not in 13 healthy controls. Interestingly, no counterpart of this mutation has been reported in humans or other mammals, where most disease alleles map to the DNA-binding domain or, more rarely, the oligomerization domain or downstream hydrophobic repeat. The R377W substitution instead mapped to a highly-conserved segment between the isoform-specific region found in HSF4b and the downstream hydrophobic region. This mutation is unlikely to affect DNA binding or trimer formation, so we predict that it influences the interaction between HSF4 and its binding partners. The identification of this mutation reveals a potential new genetic determinant that will could to predict the risk of cataracts in giant pandas.

## Materials and methods

### Proband and other samples

The proband in this study is Xinxing (S4, Table S1), a female giant panda born in 1982 in Bao-xing County (Western margin of Sichuan Basin, Ya’an, China). Xinxing began to develop cataracts in 2010 and now also shows signs of corneal atrophy. She has poor vision and slow movement. Her parents came from the wild and suffered from hypertension, but it was not possible to ascertain their ages at death and it is also unknown if they were developing cataracts at this time. Xinxing was selected as a proband for DNA sequencing along with 14 unrelated captive giant pandas for comparison representing five geographically diverse regions of China—Beijing, Baoxing, Ya’an, Wolong, and Chengdu (Table S1). We drew 2 ml of blood for each sample during a daily physical examination (without anesthetic) and the samples were initially stored at − 80 °C. The samples were collected in accordance with the Wildlife Protection Law of the People’s Republic of China (President of the People’s Republic of China No. 16), and the sampling procedure and subsequent experiments were approved by the Beijing Zoo Academic and Ethics Committee.

### Mutation detection

We selected 11 candidate genes known to be associated with cataracts in mammals: *CRYAB*, *CRYGC*, *CRYBB1*, *CRYBA1*, *HSP/GC/B6*, *HSP/GC/B7*, *HSP/GC/B9*, *GJA3*, *AQP3*, *MIP* and *HSF4*. Genomic DNA was extracted from thawed blood samples using the phenol–chloroform method (EMD Millipore/Sigma-Aldrich) and PCR was carried out using the exon-spanning primers listed in Table S2. Each 25-µl reaction comprised 1.5 mM MgCl_2_, 0.2 mM dNTPs, 0.5 μM of the appropriate forward and reverse primers, 2.5 U Taq DNA polymerase (TianGen) and 20 ng genomic DNA in 1 × PCR buffer (TianGen). The samples were denatured at 95 °C for 5 min, followed by 34 cycles of denaturation at 95 °C for 30 s, annealing at 57–63 °C (depending on the primer pair) for 30 s, and extension at 72 °C for 30 s, and a final extension step at 72 °C for 10 min. The products were sequenced using an ABI 3730 Automated Sequencer (PE Biosystems), analyzed using Chromas v2.33, and compared to the reference sequence in the NCBI database.

### Bioinformatics analysis

HSF4 orthologs from several mammalian species were aligned using CLC Free Workbench v4.5.1 (CLC Bio, Aarhus, Denmark). Protein hydrophilicity/hydrophobicity was determined using ProtScale^41^. The effects of amino acid substitution on the structure of HSF4 and interactions between amino acid side chains were predicted using THREADER v3.5^42,43^ and Modeller v9.22^44^, and were visualized using Discovery Studio Visualizer.

## Supplementary Information

Supplementary Information 1.

Supplementary Information 2.
