# Supplementary material for: A novel missense mutation in the HSF4 gene of giant pandas with senile congenital cataracts
Source: Sci Rep. 2021 Mar 8;11:5411. doi: 10.1038/s41598-021-84741-5 (PMC7940430; doi:10.1038/s41598-021-84741-5)
Supplement: Supplementary file 1 — Supplementary Information 1. [file 41598_2021_84741_MOESM1_ESM.docx]

|  | Sample | Birth year | Sex | Status | Origin | Mutation | Cataracts | Comments |
| --- | --- | --- | --- | --- | --- | --- | --- | --- |
| 1 | S1 | 1986 | Male | Dead | Beijing | c.1129C>T | + |  |
| 2 | S2 | 1996 | Female | Dead | Beijing | – | – | S1’s daughter |
| 3 | S3 | 1986 | Female | Dead | Beijing | – | – | S2’s mother |
| 4 | S4 (proband) | 1982 | Female | Alive | Baoxing | c.1129C>T | + |  |
| 5 | S5 | 2013 | Male | Alive | Beijing | – | – |  |
| 6 | S6 | 1993 | Female | Alive | Beijing | – | – |  |
| 7 | S7 | 1992 | Male | Alive | Beijing | – | – |  |
| 8 | S8 | 1999 | Male | Alive | Beijing | – | – |  |
| 9 | S9 | 2013 | Male | Alive | Beijing | – | – |  |
| 10 | S10 | 1998 | Male | Dead | Beijing | – | – |  |
| 11 | S11 | 1999 | Male | Alive | Baoxing | – | – |  |
| 12 | S12 | 1998 | Female | Alive | Wolong | – | – |  |
| 13 | S13 | 1989 | Female | Dead | Chengdu | – | – |  |
| 14 | S14 | 2009 | Female | Alive | Ya’an | – | – |  |
| 15 | S15 | 2011 | Female | Alive | Ya’an | – | – |  |

**Table S1. Characteristics of the proband and unrelated specimens.**
