# Supplementary material for: A novel missense mutation in the HSF4 gene of giant pandas with senile congenital cataracts
Source: Sci Rep. 2021 Mar 8;11:5411. doi: 10.1038/s41598-021-84741-5 (PMC7940430; doi:10.1038/s41598-021-84741-5)
Supplement: Supplementary file 2 — Supplementary Information 2. [file 41598_2021_84741_MOESM2_ESM.doc]

**Table S2 Candidate genes associated with congenital cataracts in humans and other mammals**

| Gene name | Primer | Forward (5′-3′) | Reverse (5′-3′) | Temperature（℃） |
| --- | --- | --- | --- | --- |
| CRYAB | CRYAB-1 | CCTGACTCCGTTCCAACTCC | CGGGCATCCTAATTAGAAGTTAGGGG | 60 |
|  | CRYAB-2 | GTCTGATGACCACATGGTAT | CCACTGGCTTAAAAATGGGA | 53 |
|  | CRYAB-3 | ATTTCAGATTTCTCAGACTCTCATT | AAGGACTAGAAAGGTTAAGTGAC | 53 |
| CRYBA1 | CRYBA1-1 | CTGAGATCAAGACCACAGCCTCCGA | ATTTCTTGGAGCCCCACGCAC | 60 |
|  | CRYBA1-2 | TGGGCGGCAGCTTACCTGCAT | TTCCACCCATCGCGGGCTTCC | 60 |
|  | CRYBA1-3 | CATCCCTAGCCTGGGCATGTA | GCCCCCAAAGCCAGTACCAAG | 60 |
|  | CRYBA1-4 | ATTACATTTACAAACCTAGTAACGT | CTCTTTTAGCTTAATGAAAATACCT | 51 |
|  | CRYBA1-5 | AAAGGCATTCTATTCTGTACCAATGGTG | TTCGACTCTTGGCTCAGGGCATG | 60 |
| CRYBB1 | CRYBB1-1 | GGACCGAGGGGTGGATTTGT | TCGCAGCCTTCTTTGAACTCATT | 58 |
|  | CRYBB1-2 | AGGCCTGGAGGAAGGCATTTCA | GTACCTCTAGGGAGGCTGGTGT | 60 |
|  | CRYBB1-3 | GCTGTGCAGGACTGCTTTTATTT | ATAATACCTCCCTCCTGAGCTTG | 55 |
|  | CRYBB1-4 | TAACTGGGGAAACATTTCGCTCACA | AAACCAGGGGCTCATGTTCTCATTT | 57 |
|  | CRYBB1-5 | ATTACTTCATTGAATCGTCATGACA | TTTAGAACCAATTATCCACGTTTGT | 53 |
|  | CRYBB1-6 | CCCCGTAATAAACCAGCCCTAGTGACC | AGCTGCCTTCCCCTGAGATCATG | 60 |
| CRYGC | CRYGC-1 | ACTGAATGCAGCACGTAAAAGGG | TGTGCATTTCTGTACCTGGGCTC | 58 |
|  | CRYGC-2 | AATGCAAACACGCTTTACATGCC | CCTCCCCACTTATTTGCTCCTCA | 58 |
| HSPB6 | HSPB6-1 | CGCGCCTCAGCCCCGTTGCC | CCTCCTTCAGACTCCCCAACGCTCCC | 60 |
|  | HSPB6-2 | CAGGGGAAGGGGGTCTCATCACT | CCCGCCCCGTTCTGGCCCCAC | 57 |
|  | HSPB6-3 | CCAGAACGGGGCGGGGCATC | ACAGCTTTAGCACATTTATTGGGACAAC | 57 |
| HSPB7 | HSPB7-1 | CTTGGCGTCTCGAATCCTGCTTGGTC | AGACAATACCGGAATCCCCTCTCTCA | 55 |
|  | HSPB7-2 | CCAGCCACACGCCAGCCCCTTTATGT | CTGGAGCCAAAGACTGGAGTTTGGGA | 57 |
|  | HSPB7-3 | GTGTGTGCGGGACTGTCAGCAGTG | CTGACCTCTGGGGCCACAACCGTT | 60 |
|  | HSPB7-4 | CACACAGTGTCACGCAGCCCACCT | TGGCTCCTAAAGAAAGGATCTCAGTGC | 60 |
|  | HSPB7-5 | AGCCCCTCTGCACCTGAGACTAGA | GCTGGCTTGGGGCTCAGGGAGTG | 60 |
| HSPB9 | HSPB9 | ATGCAGCGGGTCGGTAGCGG | TCCATTATGCCTCAACCTCTGACCCCAACC | 60 |
| GJA3 | GJA3-1 | GCTGCGGACGTTTATCTAA | GGGGAGTCTGAGCGATAGT | 55 |
|  | GJA3-2 | CTTCCTGTACGGCTTTGAG | TGGGTTGTTGTGAATACCT | 53 |
|  | GJA3-3 | TCCAGACAGCTTTATAATA | AACAAACAGCGACTCAGAT | 50 |
| AQP3 | AQP3-1 | CACCATTGCCTCTCAGACCCG | GAGCTGTCAGTGGGAGTAACT | 59 |
|  | AQP3-2 | AAATTAAAGTTTGGGCATTGT | TGATAATCGGATGCCAAGGTG | 51 |
|  | AQP3-3 | AGAAACTTGACACTTTGAACC | ACGTGACTGCTGTATTACTCA | 53 |
|  | AQP3-4 | GCTGCCCACCCTCCAAAGACT | TTCCCGTGTTCAGCCCTTTCC | 60 |
|  | AQP3-5 | CTTAACAAAACAGGAATTGGC | CTTCACATTCTCCTGCTCGTT | 54 |
|  | AQP3-6-1 | TCCCCGCCTTTTCACCGCCAT | AGACCCCTCGCACTTTCCTCA | 60 |
|  | AQP3-6-2 | ACGAGCAGTGGGTGTGTGAGC | GTGGGGCCGCCGAGTTCAAGC | 60 |
| MIP | MIP-1 | CTCTGCTTCTCTCCCAGT | GAACAAAAGCTGAAACCA | 51 |
|  | MIP-2 | TGGTTTCAGCTTTTGTTC | AGACAAACTGGATAGGGG | 50 |
|  | MIP-3 | TATTCCTCTCTTCTGTGA | ATAATGTGCTAGAACTGG | 50 |
|  | MIP-4 | ACACTGGTTTCTGCTTAA | AACACACACATAAATTAA | 50 |
| HSF4 | HSF4-1 | GCGTACGGGTGATAACTCC | CCATCTCACACACCCCCTC | 58 |
|  | HSF4-2 | CTTGTCCCATGTCTCCAGG | CAGAATGGTAGGTCTAAAC | 54 |
|  | HSF4-5-6 | GACCGCTCCTTCCTCTCTCCT | ATTGGGGGGAGAGGGAAAGTC | 60 |
|  | HSF4-7 | CCCTGTGTTCCCAAATTTTCC | TAAGGAGTAAGGTTTGAGGGG | 55 |
|  | HSF4-8-9 | TCCTTTCCCCCATGTCCCTAA | TTGACCTGATGGGAGTTGCTA | 56 |
|  | HSF4-10-12 | ATCCCAAGACCCCAGTTCCAT | ATCCATTCTCAACCTGCCCAC | 57 |
|  | HSF4-13 | TTAAGCTACCCATCTTCTCGA | TGAACCCTCTCTGCTTATCCG | 56 |
